# Differences in weight status and energy-balance related behaviors among schoolchildren in German-speaking Switzerland compared to seven countries in Europe

**DOI:** 10.1186/1479-5868-9-139

**Published:** 2012-11-29

**Authors:** Michael Herzig, Alain Dössegger, Urs Mäder, Susi Kriemler, Tina Wunderlin, Leticia Grize, Johannes Brug, Yannis Manios, Charlotte Braun-Fahrländer, Bettina Bringolf-Isler

**Affiliations:** 1 Swiss Tropical and Public Health Institute, Basel, Switzerland; 2 University of Basel, Basel, Switzerland; 3 Swiss Federal Institute of Sport, Magglingen, Switzerland; 4 Department of Epidemiology and Biostatistics and EMGO Institute for Health and Care Research, VU University Medical Center, Amsterdam, The Netherlands; 5 Department of Nutrition and Dietetics, Harokopio University, Athens, Greece

**Keywords:** Schoolchildren, Overweight, Diet, Physical activity, Sedentary behavior

## Abstract

**Background:**

Overweight in children and adolescents have increased significantly and are a major public health problem. To allow international comparisons, Switzerland joined the European study ‘ENERGY’ cross sectional survey consortium that investigated the prevalence of overweight and obesity as well as selected dietary, physical and sedentary behaviors of 10–12 years old pupils across seven other countries in Europe. The aims of the present study was to compare body composition and energy-balance related behaviors of Swiss schoolchildren to those of the seven European ENERGY-countries and to analyze overweight and energy-balance related behaviors of Swiss children according to socio-demographic factors.

**Methods:**

A school-based cross-sectional study among 10–12 year old children was conducted in Switzerland and seven other European countries using a standardized protocol. Body height, weight and waist-circumference were measured by trained research assistants. Energy-balance related behaviors –i.e. selected dietary, physical activity and screen-viewing behaviors were assessed by questionnaires. Weight status and behaviors in Switzerland were compared to the seven European ENERGY countries. Within the Swiss sample, analyses stratified by gender, parental education and ethnicity were performed.

**Results:**

Data of 546 Swiss children (mean age 11.6±0.8y, 48% girls) were obtained and compared to the ENERGY- results (N=7.148; mean age 11.5±0.8y, 48% girls). In Switzerland significantly less children were overweight (13.9%) or obese (2.3%) compared to the average across the ENERGY-countries (23.7% and 4.7%, respectively), and were even somewhat lower than the ENERGY countries with the lowest prevalence. Sugar sweetened beverage intakes and breakfast habits of Swiss children did not differ significantly from those of ENERGY. However, the mean time devoted by Swiss children to walking or cycling to school and attending sports activities was significantly higher and screen time significantly lower compared to the other ENERGY-countries. Within the Swiss, sample relatively large and consistent differences were observed between children from native and non-native ethnicity.

**Conclusions:**

The prevalence of overweight and obesity among Swiss children are substantial but significantly lower compared to all other European ENERGY-Partners, probably due to the fact that Swiss children were found to be more active and less sedentary comparing to the rest of the European sample.

## Background

The number of overweight children in Europe has increased substantially over the last decades [1]. Although a recent meta-analysis indicated that this increase might have come to an end [2,3], the prevalence of overweight children remains high and constitutes a major public health problem. Overweight and obesity in childhood and adolescence increase the likelihood of being overweight in adulthood [4] and are associated with an increased risk for various diseases, such as type II diabetes, chronic back pain or cardiovascular diseases [5]. Thus they are important determinants of avoidable burden of disease.

Recent reviews suggested increased consumption of sugar sweetened beverages, breakfast skipping, lack of physical activity, high levels of TV and computer time and short sleep duration to be associated with overweight and obesity among school-aged children [6-10]. The European ENERGY project “**E**uropea**N E**nergy balance **R**esearch to prevent excessive weight **G**ain among **Y**outh” has set out to develop a comprehensive intervention aiming to promote dietary and physical activity behaviors that contribute to a healthy energy balance among school-aged children [11]. A key point of the project was a cross-European study on measured weight status and reported energy-balance related behaviors (EBRB) among 10–12 year olds living in seven different European countries [11-13]. Switzerland has been invited to join the ENERGY consortium with its own funding after the ENERGY-project was approved. So far the only internationally comparable data on EBRB and overweight of Swiss children originated from the HBSC study and showed conflicting results [14]. On one hand Switzerland ranked low with respect to the prevalence of overweight and obesity and on the other levels of physical activity were also reported to be low [14]. As weight and height of the HBSC study was based on self-reports of the children and no objective assessment of physical activity was available. The ENERGY project offered the opportunity to compare overweight rates and EBRB of Swiss children to their European peers using a standardized protocol and including objectively assessed data [15].

The present analysis aims (1) to compare body mass index (BMI), waist circumference, percentages of overweight and obesity and EBRB of Swiss schoolchildren to those of the seven European ENERGY-countries and (2) to analyze overweight and EBRB of Swiss children according to socio-demographic factors.

## Methods

### Sampling and organization of the study

The school-based cross-sectional survey included anthropometric measurements, a child questionnaire and a parent questionnaire and was carried out among 10–12 year old pupils at school. A detailed description of the rationale and organization of the ENERGY-project [11] and a comprehensive description of the design, procedures of the ENERGY school-based cross-sectional survey have been published elsewhere [12]. In brief: the study was conducted between June and December 2010 in differently urbanized regions of German-speaking Switzerland. The three regions randomly selected from each of the lowest, mid and highest tertiles of degree of urbanization were Basel, St. Gallen and Bern/Solothurn. The schools in these regions were randomly selected for inclusion in the study. The ENERGY protocol aimed for a minimum sample of 1,000 schoolchildren per country and one parent/caretaker for each child.

A school recruitment letter was sent to the headmaster of the sampled schools, followed by a personal telephone call. Following the school’s agreement, parents received a letter explaining the purpose of the study and were asked for written consent for their child’s and own participation. The study protocol was approved by the ethics committees of the participating cantons (Basel, Bern, Aargau and St. Gallen).

### Measurements

Measurements were conducted according to the standardized ENERGY protocols. The children comple-ted questionnaires and anthropometric measurements during school time. The parent/caretaker filled in the questionnaire at home. Detailed information regarding the procedures, training of research staff, development of questionnaires, are published elsewhere [12].

### Anthropometric measurements

Body height, weight and waist-circumference were measured by trained research assistants. The children were measured in light clothing without shoes. Body height was measured with SECA 225 Leicester Portable stadiometer (accuracy of 0.1 cm). Weight was measured with a calibrated electronic scale SECA 861 (accuracy of 0.1 kg), waist circumference with the SECA 201 measuring band (accuracy 0.1 cm). Two readings of each measurement were obtained and the mean was used for analyses. When the two readings differed more than 1%, a third measurement was conducted.

Body mass index (BMI) was calculated as BMI=weight/height^2^ (Kg/m^2^). Overweight status (overweight, obesity) was calculated based on the International Obesity Task Force criteria (IOTF) [16]. In order to make BMI compa-rable across age and sex, the BMI standard deviation scores (z-scores) [17] were calculated.

### Questionnaire

The English version of the ENERGY questionnaires were translated into German and then back translated and compared to the English version. Dietary habits, physical activity and screen viewing behaviors were assessed by the child questionnaire. Child’s sleep duration, parental education and ethnic background were reported by the parents.

#### Dietary habits

Intake of soft drinks and fruit juices were each assessed with two food frequency questions (FFQ) referring to a general week and to the last 24-hrs. First, children were asked on how many days per week they drank the beverage. Subsequently they were asked to indicate how much they drank on days they consumed the beverage by ticking the number of glasses or bottles, which were pictured in the questionnaire. Mean intake in milliliters per day was calculated from the FFQ by multiplication of number of days per week and amount per day in ml divided by 7.

#### Breakfast habit

Were assessed by two questions asking the children on how many schooldays per week and on how many weekend days they normally had breakfast. The frequency score was recoded into a skipping breakfast score (had breakfast 7 days/week; had breakfast 0–6 times/week).

#### Physical activity behaviors

Transport to school was assessed by two questions on how many days per week the child cycled and or walked to school and two questions on how long the bike ride or walk to school was. Questions referred to a general week and to the last 24hrs. Total bike/walk time per week was calculated by multiplying the number of days with the mean time of the answering category multiplied by 2 (round-trip). Total active transport to and from school was calculated by adding up total bike and walk times. Regarding organized sports participation, questions were included on how many hours per week children participated in different sports activities. Based on the answers average hours of sport participation per week was calculated for each child.

#### Sedentary behavior

Screen time was assessed by asking questions about time spent watching TV (including video and DVD) and computer activities for weekdays and weekend days sepa-rately, referring to a general week and to the last 24hrs. Mean TV, computer and total screen time per day were calculated.

#### Sleeping

Parents indicated how many hours the child sleeps on average per night, separately, for weekdays and weekends. A mean number of hours of sleep per night was then calculated.

#### Parental education

Was assessed as a measure of socio-economic background by asking parents to report their own level of education and that of the other parent/caregiver. For analyses, information of the parent/caregiver with the longer education was used. In contrast to the other ENERGY-Partners, parental education in Switzerland was dichotomized into low and high using a cut-off of 12 years of education (ENERGY-Partners 14 years) since preschool education does not count as school education in Switzerland.

#### Ethnic background

Was assessed by all ENERGY Partners based on the language spoken at home or on the country of origin of the parents [18]. It was classified as ‘non-native’ if another language than German, French or Italian was spoken at home or one or both parents were born in a foreign country, and as ‘native’ if German was spoken at home or if both parents were born in Switzerland.

#### Questionnaire validity

Test-retest reliability was tested in Switzerland according to the ENERGY protocol [19] by administrating the questionnaire one week after the first assessment to 114 school-children. To assess construct validity, the agreement between questionnaire responses and a subsequent face-to-face interview (15 children) was evaluated. Both test-retest reliability and construct validity were determined by calculating the intra-class correlation coefficient (ICC). Of the 36 questionnaire items related to EBRB in the child questionnaire 72% showed good to excellent test-retest reliability as indicated by ICCs > 0.60, whereas 22% showed moderate (ICC 0.41-0.60) and 0.5% poor reliability (≤ 0.40). Similar results were found for construct validity.

### Statistical analysis

Stata 11.2 (StataCorp LP Texas, USA) was used for all statistical analyses. Means and standard deviations for continuous variables and percentages for categorical variables were reported for anthropometric measurements and EBRB. Because of skewed distribution, medians were also provided (either in the tables or in the Additional file 1, Additional file 2, Additional file 3, Additional file 4). All skewed distributions were log-transformed for analyses. T-test was performed to assess differences in means of anthropometrics and EBRB between the Swiss sample and the ENERGY-Partners. In addition to the mean values of the ENERGY-Partners, the range of individual country means was also provided. To assess differences in anthropometrics and EBRBs within Switzerland according to gender, ethnic background and parental education t-tests for means, Wilcoxon signed-rank tests for medians and chi-squared tests for proportions were calculated. Because of the high proportion of zeroes in the variables ‘weekly minutes of walking to school’ and ‘weekly minutes of cycling to school’, we used bootstrap with 1,000 replications to confirm the results of the Wilcoxon-test [20]. For all analyses a *p*-value of 0.05 was used for statistical significance.

## Results

### Participant characteristics

**Figure 1:**
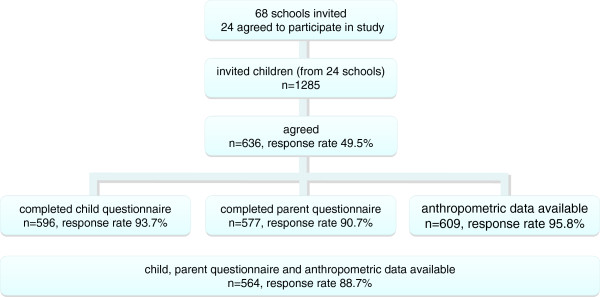
Overview of data collection and response rate in Switzerland.

24 of the 68 invited schools (35%) agreed to participate (Figure 1). Many schools declined participation arguing that they were already busy with several other surveys. Informed consent for study participation was avail-able for 636 (49.5%) out of 1286 invited children. Rates widely ranged between schools (20% to 81%). Most children completed the child questionnaire (n=596) and the anthropometric measurements (n=609), and had a parent completed questionnaire (n=577). Complete data was available for 564 children. Their mean age was 11.6±0.8yrs, and 48% were girls. The total sample of the seven ENERGY-Partners comprised 7757 children (mean age 11.5±0.8yrs, 48% girls).

### Anthropometrics

A significantly lower prevalence of overweight (13.9%) and obese (2.3%) children were observed in Switzerland when compared to the mean of the seven European ENERGY-countries (23.7% and 4.7%, respectively) (Table 1). The overweight prevalence differed greatly between the seven European ENERGY countries (ranging from 14.4% in Norway to 40.8% in Greece) but was always higher than in Switzerland. Similarly, mean BMI and mean waist circumference were lower in Swiss children.

**Table 1:** Anthropometrics of the Swiss sample and the ENERGY-Partners

**Anthropometrics**	**Switzerland**	**ENERGY-Partners**	
	**Total**	**Total**	**Range**
	**N=609**	**N=7148**	
BMI^a^ (mean, SD^b^)	18.0 (2.8)***	19.1 (3.3)	18.2; 20.4
BMI^b^ (median)	17.4 (16.1;19.3)***	18.4 (16.7;20.8)	17.5; 19.8
WC^c^ (mean, SD^b^)	63.5 (7.5)***	66.2 (8.9)	63.3; 70.5
WC^c^ (median)	62.2 (58.5;67.1)***	64.2 (60.0;70.8)	61.5; 69.0
% Overweight^d^	13.9***	23.7	14.4; 40.8
% Obese^d^	2.3**	4.7	1.4; 10.4
% BMI 1SD^e^	18.4***	30.5	19.5; 49.3
% BMI 2SD^e^	5.0***	9.7	4.0; 20.7
% BMI 3SD^e^	0.2*	0.8	0.1; 2.0

^a^BMI=body mass index.

^b^SD=standard deviation.

^c^WC=waist circumference.

^d^overweight categories based on the IOTF criteria, including both overweight and obesity.

^e^BMI standard deviation scores, also called BMI z-scores, are measures of relative weight adjusted for child age and sex.

*p≤0,05.

**p≤0,01.

***p≤0,001.

Within the Swiss sample, overweight and obesity rates (and related anthropometric indices) were significantly lower in Swiss natives as compared to non-natives most notably when ethnicity was based on language spoken at home (Table 2) . Gender and socio-economic differences were less pronounced.

**Table 2:** Anthropometrics of the Swiss sample stratified by gender, parental education and ethnicity

**Anthropometrics**	**Total**	**Gender**		**Parental education**		**Language spoken at home**		**Country of birth of parents**	
		**Boys**	**Girls**	**Low**	**High**	**Non-native**	**Native**	**Non-native**	**Native**
	**N=609**	**N=311**	**N=285**	**N=175**	**N=381**	**N=139**	**N=455**	**N=201**	**N=359**
BMI^a^ (mean, SD^b^)	18.0 (2.8)	18.1 (2.7)	17.9 (2.8)	18.3 (3.1)*	17.8 (2.6)	18.5 (3.2)*	17.9 (2.6)	18.2 (3.1)	17.8 (2.5)
BMI^a^ (median)	17.4	17.4	17.4	17.7	17.3	17.9	17.3	17.5	17.2
WC^c^ (mean, SD^b^)	63.5 (7.5)	64.6 (7.5)***	62.4 (7.3)	64.3 (8.1)*	62.9 (7.0)	65.9 (8.5)***	62.9 (7.1)	64.8 (8.1)***	62.5 (6.8)
WC^c^ (median)	62.2	63.1	61.5	63.3	61.6	64.0	61.8	63.0	61.4
% Overweight^d^	13.9	15.8	11.6	15.4	12.6	21.6**	11.5	15.4	12.3
% Obese^d^	2.3	1.9	2.5	3.4	1.6	5.8***	1.1	4.5*	0.8
% BMI 1SD^e^	18.4	21.6*	14.7	20.6	16.8	25.2*	16.3	20.9	16.2
% BMI 2SD^e^	5.0	6.5	3.2	5.7	4.5	7.9	4.0	7.0	3.6
% BMI 3SD^e^	0.2	0.3	0.0	0.6	0.0	0.0	0.2	0.5	0.0

^a^BMI=body mass index.

^b^SD=standard deviation.

^c^WC=waist circumference.

^d^overweight categories based on the IOTF criteria, including both overweight and obesity.

^e^BMI standard deviation scores, also called BMI z-scores, are measures of relative weight adjusted for child age and sex.

*p≤0,05.

**p≤0,01.

***p≤0,001.

### Dietary behaviors

Mean consumption of soft-drinks of Swiss children (388 ml/day) differed significantly from those of the European ENERGY-Partners (Table 3) but were well within their range (soft drink consumption ranging from 114 ml/day in Greece to 632 ml/day in the Netherlands). The consumption of fruit juice (314 ml/day) was slightly but not significantly higher in Switzerland compared to the European average. Within Switzerland, boys from lower socio-economic background and non-native reported a significantly higher intake of soft drinks and fruit juices than girls and children from higher socio-economic background and Swiss natives (Table 4).

**Table 3:** Dietary habits, physical activity and sedentary behaviors of the Swiss sample and the ENERGY-Partners

	**Switzerland**	**ENERGY-Partners**	**Range**
	**Total**	**Total**	**between ENERGY-Partners**
	**N=596**	**N=7194**	
**Dietary habits**			
Soft drink FFQ (ml/day)	388 (522)*	348 (495)	114; 632
Soft drink 24h recall (ml/day)	390 (464)**	359 (480)	154; 643
Fruit juice FFQ (ml/day)	314 (383)	295 (351)	187; 384
Fruit juice 24h recall (ml/day)	290 (401)	278 (350)	152; 391
Breakfast (days/week)	6.0 (1.7)	5.9 (1.8)	5.1; 6.7
Skipped breakfast ≥ 1/week (%)	32.4	33.7	14.4; 51.6
**Physical activity behavior**			
Total active commuting (days/week)	4.7 (1.5)***	3.9 (2.4)	2.4; 6.1
Total active commuting (min/week)	71 (54)***	56 (57)	40; 103
Active commuting 24h recall (min/day)	13.7 (11.4)***	8.5 (10.0)	5.4; 13.1
Cycling to school (days/week)	1.2 (2.0)	1.3 (2.0)	0.1; 3.3
Cycling to school (min/week)	20.0 (41.8)	17.6 (36.4)	0.5; 47.6
Walking to school (days/week)	3.5 (2.0)***	2.6 (2.3)	1.3; 4.0
Walking to school (min/week)	51.0 (51.0)***	38.0 (47.2)	13.6; 59.9
Sport participation (min/week)	164 (105)***	149 (105)	128; 173
**Sedentary behavior**			
Screen time FQ (min/day)	131 (90)***	193 (103)	176; 213
Screen time 24h recall (min/day)	74 (74)***	124 (96)	105; 147
TV time FQ (min/day)	79 (54)***	112 (61)	101; 123
TV time-24h recall (min/day)	49 (50)***	78 (60)	67; 94
Computer time FQ (min/day)	53 (49)***	81 (61)	73; 94
Computer time 24h recall (min/day)	25 (42)***	46 (58)	35; 59
Sleep duration (hr/night)	9.5 (0.7)***	9.2 (0.8)	8.7; 9.7

*p≤0,05.

**p≤0,01.

***p≤0,001.

**Table 4:** Dietary habits of the Swiss sample stratified by gender, parental education and ethnicity

**Dietary habits**	**Total**	**Gender**		**Parental education**		**Language spoken at home**		**Country of birth of parents**	
		**Boys**	**Girls**	**Low**	**High**	**Non-native**	**Native**	**Non-native**	**Native**
	**N=596**	**N=311**	**N=285**	**N=175**	**N=381**	**N=139**	**N=455**	**N=201**	**N=359**
Soft drink FFQ (ml/day)	388 (522)	461 (540)***	307 (447)	496 (567)***	311 (458)	493 (621)**	355 (484)	403 (507)	351 (499)
Soft drink 24h recall (ml/day)	390 (464)	456 (520)**	318 (434)	464 (507)***	331 (412)	495 (480)***	359 (456)	423 (431)**	355 (472)
Fruit juice FFQ (ml/day)	314 (383)	360 (431)**	263 (315)	314 (381)	294 (354)	473 (512)***	265 (318)	383 (456)**	259 (309)
Fruit juice 24h recall (ml/day)	290 (401)	338 (421)**	239 (372)	271 (332)	285 (407)	383 (476)*	261 (371)	340 (430)**	253 (379)
Breakfast (days/week)	6.0 (1.7)	5.9 (1.8)	6.1 (1.6)	5.9 (1.8)	6.1 (1.7)	5.7 (1.9)*	6.1 (1.7)	5.9 (1.7)	6.2 (1.7)
Skipped breakfast ≥ 1/week (%)	32.4	35.1	29.4	37.5	29.2	39.6*	30.1	37.4*	28.0

*p≤0,05.

**p≤0,01.

***p≤0,001.

### Physical activity

Time spent with active commuting (walking and biking to school combined) was significantly higher in Switzerland compared to the average time spent by the European ENERGY-Partners (Table 3). The difference resulted mainly from the higher number of days the children walked to school and the longer duration of the walking trips. However, the total active commuting time in Switzerland was within the range of the European partners (40 min/week in Greece and 103 min/week in Norway).

Swiss children also reported to be significantly longer engaged in sports activities (164 minutes/week) than the European children on average (149 minutes/week) although the Swiss results were again within the range of the ENERGY-Partners (ranging from 128 minutes per week in Greece to 173 minutes per week in Norway).

Within Switzerland, boys reported significantly more minutes of engagement in sports (190 minutes/week) than girls (135 minutes/week) (Table 5). Non-native Swiss children (mainly when ethnicity was based on the country of origin of their parents) spent significantly less time commuting actively to school, mainly because of less time spent for cycling. Girls tended to walk more often to school.

**Table 5:** Physical activity behaviors of the Swiss sample stratified by gender, parental education and ethnicity

**Physical activity behavior**	**Total**	**Gender**		**Parental education**		**Language spoken at home**		**Country of birth of parents**	
		**Boys**	**Girls**	**Low**	**High**	**Non-native**	**Native**	**Non-native**	**Native**
	**N=596**	**N=311**	**N=285**	**N=175**	**N=381**	**N=139**	**N=455**	**N=201**	**N=359**
Total active commuting (days/week)	4.7 (1.5)	4.6 (1.6)	4.8 (1.4)	4.6 (1.6)	4.7 (1.5)	4.6 (1.8)	4.7 (1.4)	4.6 (1.8)	4.7 (1.3)
Total active commuting (min/week)	71 (54)	68 (52)	74 (55)	70 (54)	73 (53)	64 (51)	73 (54)	62 (49)***	78 (56)
Active commuting 24h recall (min/day)	13.7 (11.4)	12.7 (10.8)*	14.7 (12.0)	12.9 (11.0)	14.6 (11.9)	11.4 (10.4)**	14.3 (11.6)	11.0 (9.6)***	15.6 (12.4)
Cycling to school (days/week)	1.2 (2.0)	1.3 (2.0)	1.2 (1.9)	1.1 (1.9)	1.3 (2.0)	1.0 (1.8)	1.3 (2.0)	0.9 (1.7)**	1.4 (2.1)
Cycling to school (min/week)	20.0 (41.8)	19.6 (39.8)	20.4 (44)	20.1 (46.8)	21.0 (41.3)	15.6 (36.4)	21.0 (42.5)	13.1 (31)**	24.9 (47.9)
Walking to school (days/week)	3.5 (2.0)	3.4 (2.1)	3.7 (2.0)	3.6 (2.0)	3.5 (2.1)	3.6 (1.9)	3.5 (2.1)	3.7 (1.9)	3.4 (2.1)
Walking to school (min/week)	51 (51)	49 (49)	54 (52)	50 (48)	52 (52)	49 (43)	52 (53)	49 (45)	53 (55)
Sport participation(min/week)	164 (105)	190 (105)**	135 (98)	164 (109)	164 (102)	165 (116)	164 (102)	159 (107)	167 (102)

*p≤0,05.

**p≤0,01.

***p≤0,001.

### Sedentary behavior and sleeping

Swiss children spent significantly less time on total screen activities (107 min/day), both for watching TV or computer activities than their European peers (Table 3). The mean time watching TV in Switzerland (79 min/day) and time spent with computer activities (53 min/day) was well below the range of time reported by the different ENERGY-Partners (ranging from 101 min/day TV time in Norway to 123 minutes/day in Greece, and from 73 min/day computer activities in Spain to 94 minutes/day in Hungary).

Within Switzerland, boys from lower socio-economic background and non-native children spent significantly more time with screen activities than their counterparts (Table 6). Gender differences were more pronounced for computer activities whereas differences with respect to socio-economic background and ethnicity were observed for both TV and computer activities (Table 6).

**Table 6:** Sedentary behaviors of the Swiss sample stratified by gender, parental education and ethnicity

**Sedentary behavior**	**Total**	**Gender**		**Parental education**		**Language spoken at home**		**Country of birth of parents**	
		**Boys**	**Girls**	**Low**	**High**	**Non-native**	**Native**	**Non-native**	**Native**
	**N=596**	**N=311**	**N=285**	**N=175**	**N=381**	**N=139**	**N=455**	**N=201**	**N=359**
Screen time FQ (min/day)	131 (90)	146 (95)***	116 (82)	150 (96)**	120 (85)	166 (109)***	121 (81)	150 (98)***	119 (84)
Screen time 24h recall (min/day)	74 (74)	87 (77)***	60 (67)	90 (84)*	65 (65)	97 (94)**	67 (65)	87 (84)*	65 (58)
TV time FQ (min/day)	79 (54)	83 (54)	75 (53)	93 (59)***	72 (51)	99 (64)***	73 (49)	91 (60)***	72 (50)
TV time-24h recall (min/day)	49 (50)	55 (50)**	42 (48)	59 (55)**	44 (46)	62 (60)*	45 (46)	55 (54)	45 (47)
Computer time FQ (min/day)	53 (49)	63 (54)***	41 (41)	57 (52)	48 (44)	68 (61)***	47 (44)	59 (62)*	47 (44)
Computer time 24h recall (min/day)	25 (42)	33 (47)***	18 (34)	31 (50)*	21 (35)	35 (52)**	22 (38)	32 (49)**	20 (34)
Sleep duration (hr/night)	9.2 (1.5)	9.5 (0.8)	9.5 (0.7)	9.5 (2.1)	9.5 (0.8)	9.5 (0.8)	9.6 (0.7)	9.6 (0.7)	9.5 (0.7)

*p≤0,05.

**p≤0,01.

***p≤0,001.

Mean sleep duration of Swiss children was significantly higher compared to that reported by the European ENERGY-Partners (Table 3). Within the Swiss sample, no difference was observed with respect to gender, socio-economic background and ethnicity (Table 6).

## Discussion

The prevalence of objectively assessed overweight and obesity among Swiss children was significantly lower than across the other European countries in the ENERGY-consortium. Reported physical activity and screen viewing behaviors were more favorable whereas dietary habits were similar. Within the Swiss sample, ethnicity was more strongly related to differences in overweight prevalence, dietary habits, active commuting and screen activities than parental education or gender.

The prevalence of overweight and obesity observed in the present study are in line with more recent Swiss studies based on measured weight and height [21,22] but are clearly higher than the rates of the HBSC study which were based on self-reports of the children [14]. Studies assessing time trends of childhood overweight prevalence in Switzerland based on measured weight and height documented a strong increase during the nineties of the last century [23] and a ‘leveling off’ or even decrease since the beginning of the new century [2,21-23]. This trend is in line with the results of a recent review analyzing data of 52 studies from Australia, Europe, Japan and the USA [3].

Within the Swiss sample no statistical significant gender difference in the prevalence of overweight and obesity was found, but ethnicity appeared as a strong risk factor as previously reported [21,23-25]. However, non-native Swiss children had a significantly lower BMI, waist circumference and proportion of overweight than the average across the ENERGY-countries.

In contrast to the previous report of the HBSC study [14] the present study showed that Swiss children accumulated significantly more minutes of active commuting and of participation in sports activities than the other ENERGY-Partners. These results are supported by the recently published accelerometer measurements of the ENERGY project [26] indicating that Swiss children spent significantly more minutes in moderate to vigorous physical activity than children from the other European countries. There might be several explanations for these findings. First, all schools in Switzerland are legally obligated to provide 3 physical education sessions per week and although children only spend one third of these lessons in moderate to vigorous physical activity, they significantly increase children’s accelerometer based MVPA levels during school time [27]. Secondly, there is a national sports promotion program ‘ Youth and Sport (Y+S)’ which offers optional physical education sessions after school as well as courses and sport camps for children and fosters children’s integration into a sports club [28]. Third, a vast proportion of Swiss children still commutes actively to school [29,30]. The results of the present study might even underestimate the time spent in active commuting as they are based on the ENERGY algorithm to calculate total commute time assuming two trips per day. Yet, children in Switzerland usually return home for lunch and therefore travel up to four times a day to or from school. Active commuting to school is popular in Switzerland since more than 95% of the Swiss children attend the public schools located closest to their homes [29] and there is no free school choice. The short distances facilitate walking or cycling to school. In addition, 64% of parents reported to perceive their children’s way to school to be safe [30]. Safety concerns of Swiss parents were mostly related to dangers from traffic (85%) and less often to violence and harassment (<10%) [30] contrasting reports, e.g. from the UK, where a large proportion of parents were worried about abduction or molestation [31].

Swiss children also indicated to spend less time with screen activities than their peers in the seven European ENERGY-Partners’ countries as also reported by the HBCS study [14]. Screen-viewing behaviors are usually assessed as an indicator of physical inactivity. Yet, recent comparisons with accelerometer-derived sedentary time clearly showed that self-reported TV and computer time did not correlate well with objective measures [32,33]. TV viewing may not be a good indicator of physical inactivity but it has been linked to unhealthy eating behaviors, such as lower fruit and vegetable intake, higher sugar-sweetened beverage consumption snacking and higher fast food intake [34] which in turn are related to overweight.

Interestingly EBRB of non native Swiss children were in most aspects comparable to the behaviour of Swiss native children (active transport, sleeping duration, screen activities) indicating some adoptive behaviour. An exception was cycling where non-native Swiss resembled more the one from southern Europe ENERGY Partners (Greece, Spain).

### Strength and limitations

An obvious limitation and potential source of bias of the present study is the low participation rate. First, the relatively small sample size reduced our ability to detect statistically significant differences between sub-groups. Second, overweight/ obesity and EBRB rates may be underestimated if participation in the study was dependent on overweight status or behavioral patterns. To address this question we evaluated whether the response rate in a given school was associated with the prevalence of overweight assuming that in schools with low participation rate a lower prevalence of overweight would result. Response rates in our samples varied between 20% and 81% and were subdivided into quartiles. The quartile with the lowest participation rate (1^st^ quartile) yielded an overweight prevalence of 25.5%, the 2^nd^ quartile 15.6%; the 3^rd^ quartile 7.2%; and the 4^th^ quartile a rate of 13.1%, thus giving no evidence of a systematic under-representation of overweight children due to selective participation. Third, the assessment of dietary habits, physical activity and sedentary behavior were self-reported and depended upon the respondents’ recall and ability to give correct answers. We thus evaluated the test-retest reliability and construct validity of the respective questions and found good levels of agreement similar to those of the ENERGY-Partners [19]. Finally the three regions included in the ENERGY study were all from the German speaking part of Switzerland (63.7% of the Swiss people) limiting the generalization for the whole country.

A clear strength of the present study was the use of the standardized ENERGY protocol allowing international comparisons and the inclusion of objective weight and height data.

## Conclusions

It can be concluded that the prevalence of overweight in Switzerland is substantial but lower than in to other countries across Europe. Children from Switzerland engaged less frequently in unfavorable energy balance related behaviors associated with overweight and obesity. However, considerable differences were observed within Switzerland, with children from non-native ethnicity more likely to be overweight and obese. Socio-economic and cultural aspects need be taken into account in planning preventive health interventions.

## Abbreviations

BMI: Body mass index; ENERGY: EuropeaN Energy balance Research to prevent excessive weight Gain among Youth; EBRB: Energy-balance related behaviors; FFQ: Food frequency questions; HBSC: Health Behaviour in School-aged Children; ICC: Intra-class correlation coefficient.

## Competing interests

The authors declare that they have no competing interests.

## Authors’ contributions

UM, BB, CB, AD and SK coordinated and supervised the Swiss study part. AD, BB, UM and TW were involved in the data collection. LG, MH, and BB performed the data cleaning. MH conducted the analyses and drafted the paper supervised by BB and CB. TW and BB tested the validity and the reliability of the questionnaires. JB developed the concept and design of the ENERGY Project. YM coordinated the international part of the cross-sectional study. All authors critically revised the draft versions of the manuscript, provided critical feedback and approved the final version.

## Supplementary Material

Additional file 1 **Dietary habits, physical activity and sedentary behaviors of the Swiss sample and the ENERGY-Partners.** Medians and 25-75% percentiles of dietary habits, physical activity and sedentary behaviors of the Swiss sample and the ENERGY-Partners. Click here for file

Additional file 2 **Dietary habits of the Swiss sample stratified by gender, parental education and ethnicity.** Medians and 25-75% percentiles of dietary habits for the total Swiss sample stratified for boys and girls, for children with low and high educated parents and for native and non-native children. Click here for file

Additional file 3 **Physical activity behaviors of the Swiss sample stratified by gender, parental education and ethnicity.** Medians and 25-75% percentiles of physical activity behaviors of the Swiss sample stratified for boys and girls, for children with low and high educated parents and for native and non-native children. Click here for file

Additional file 4 **Sedentary behaviors of the Swiss sample stratified by gender, parental education and ethnicity.** Medians and 25-75% percentiles of sedentary behaviors of the Swiss sample stratified for boys and girls, for children with low and high educated parents and for native and non-native children. Click here for file
